# Supplementary material for: A genome-wide association study of mammographic texture variation
Source: Breast Cancer Res. 2022 Nov 7;24:76. doi: 10.1186/s13058-022-01570-8 (PMC9639267; doi:10.1186/s13058-022-01570-8)
Supplement: Supplementary file 1 — Additional file 1: Fig. S1. Correlations between the four V assessments. Fig. S2. Quantile-quantile plots of the GWAS meta-analysis results. Fig. S3. Manhattan plots of the GWAS meta-analysis results. Fig. S4. Quantile-quantile plots of the P value of heterogeneity. Table S2. Sources of summary statistics of breast cancer risk and breast cancer risk factors for calculating genetic correlation. Table S3. Sources of summary statistics of breast cancer risk and breast cancer risk factors for the SNP-set test. [file 13058_2022_1570_MOESM1_ESM.docx]

**Figure S1.** Correlations between the four V assessments

















Correlations between the four V assessments. Spearman correlation between the two measures is shown on each plot. Red lines on the plots are the diagonal lines.

**Figure S2.** Quantile-quantile plots of the GWAS meta-analysis results

**a**


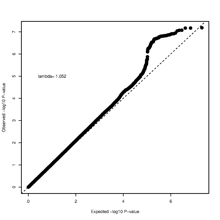

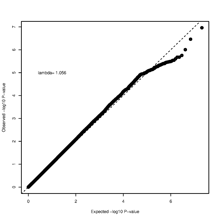

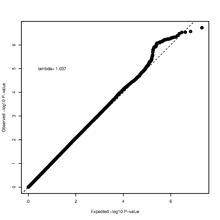

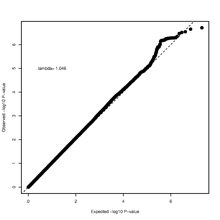

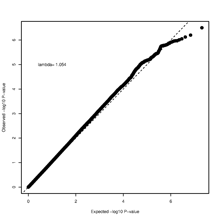

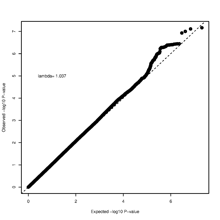


**b**

**
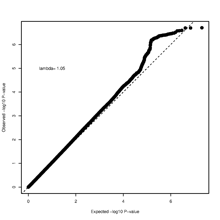

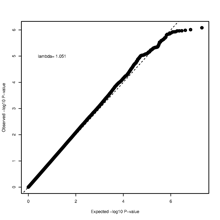

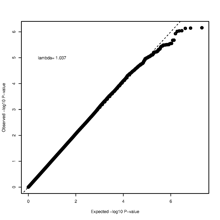

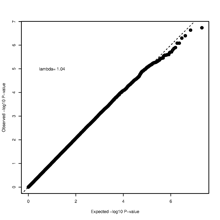

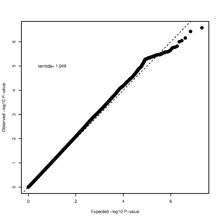

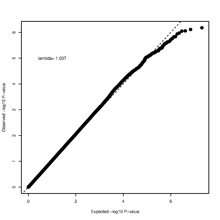
**

**c**

**
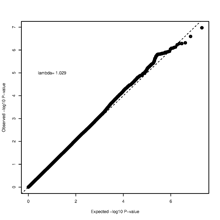

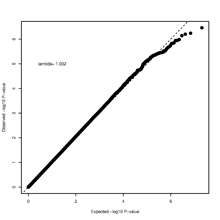

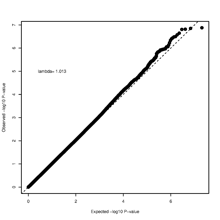

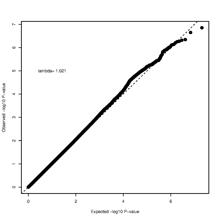

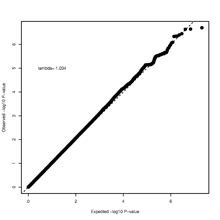

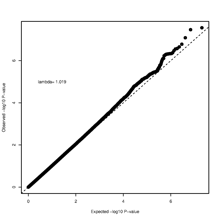
**

**d**

**
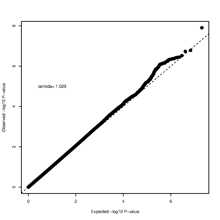

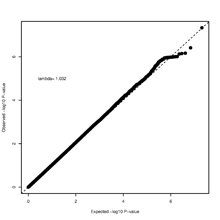

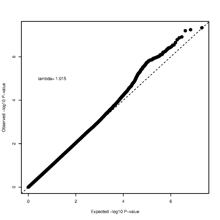

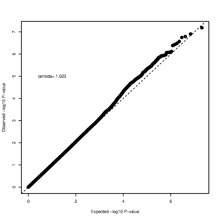

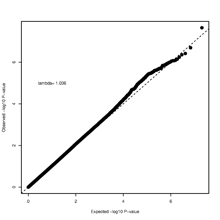

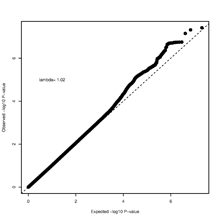
**

**e**

**
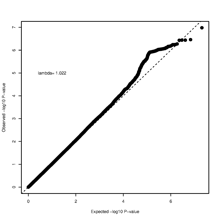

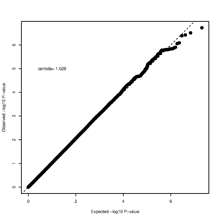

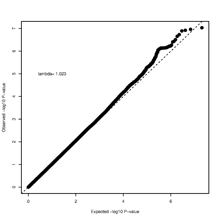

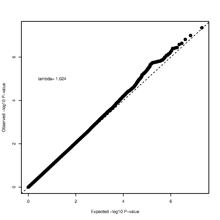

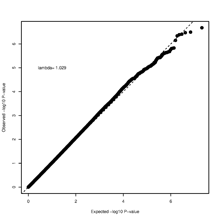

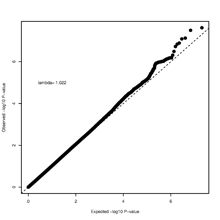
**

Quantile-quantile plots of the GWAS meta-analysis results. **a** = V65L; **b** = V75L; **c** = V65H; **d** = V75H; **e** = VSUM. On each panel, the plots corresponding to Model 0 to Model 5 from left to right.

**Figure S3.** Manhattan plots of the GWAS meta-analysis results

**a**


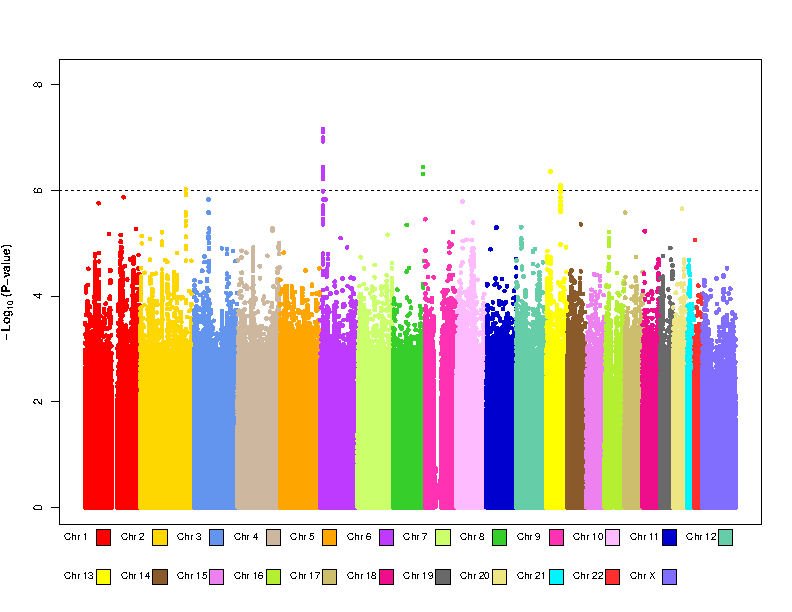

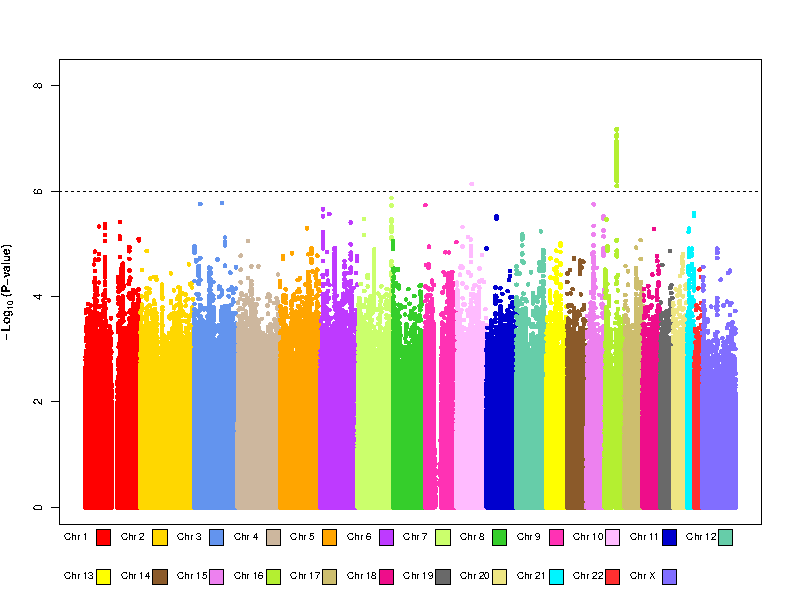

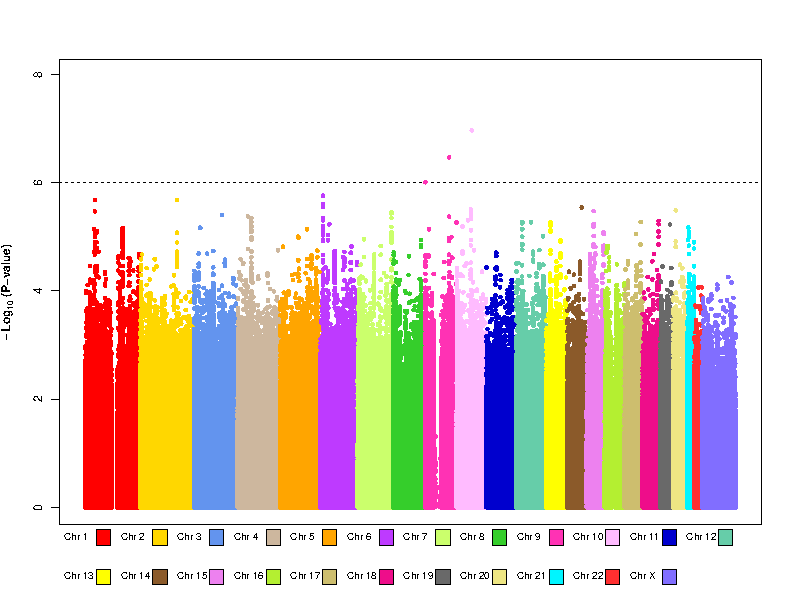

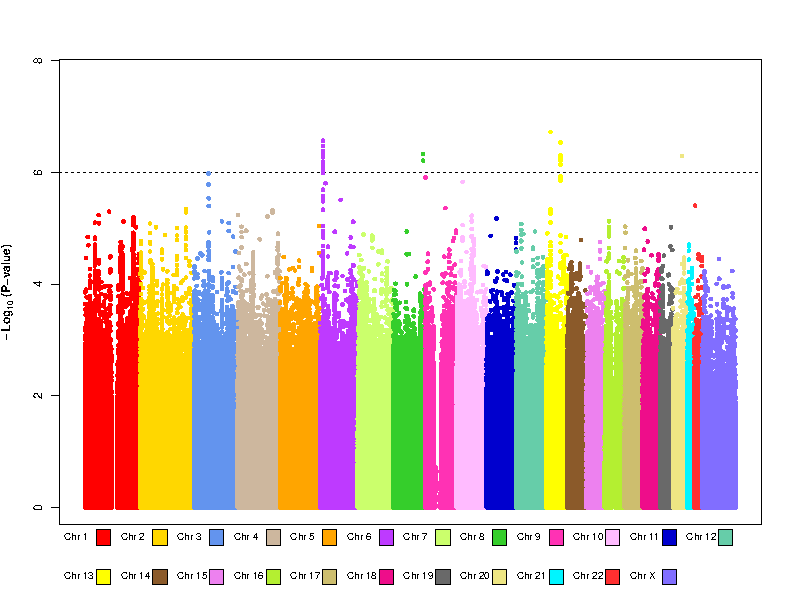

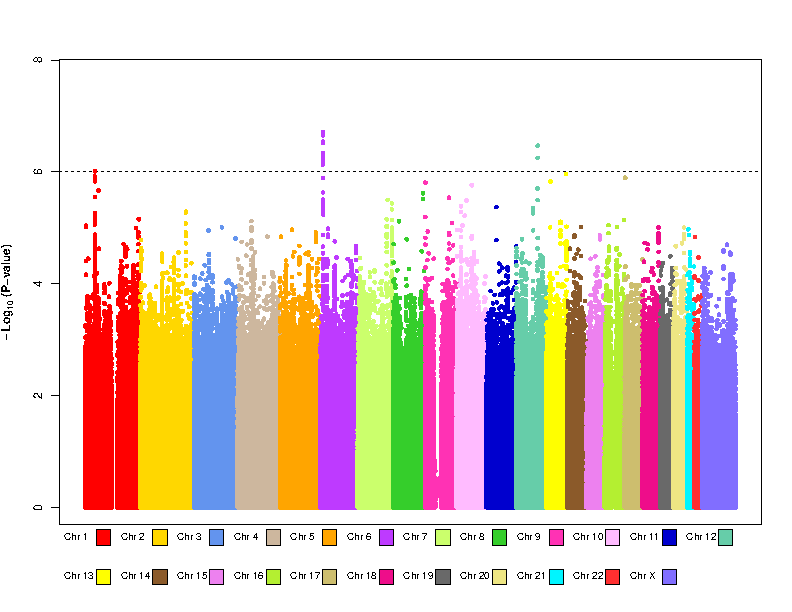

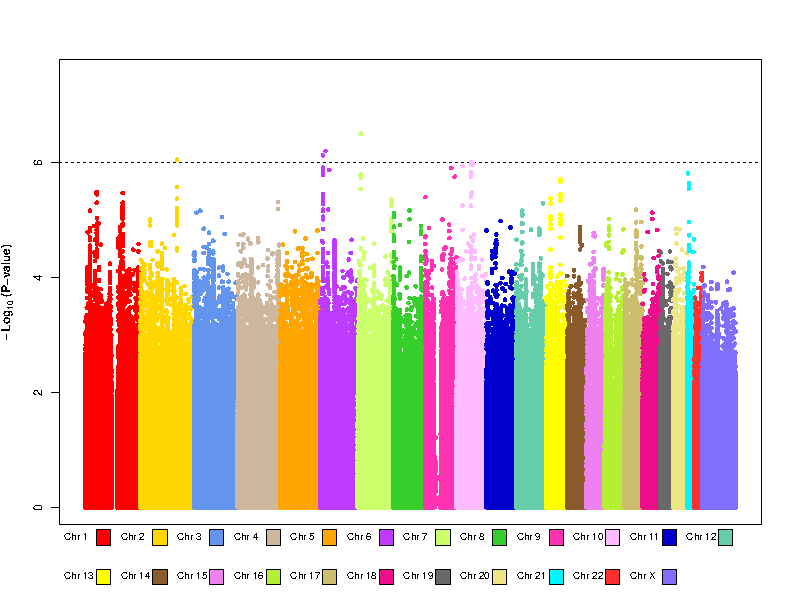


**b**


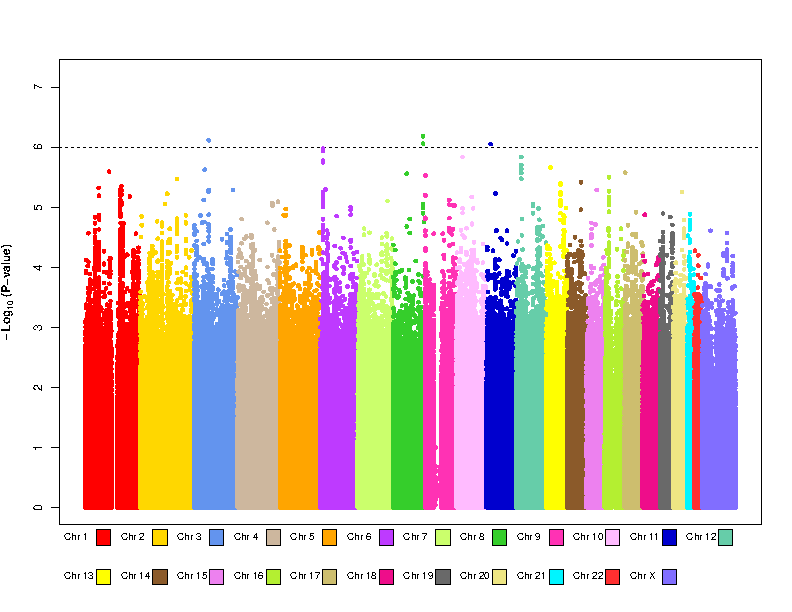

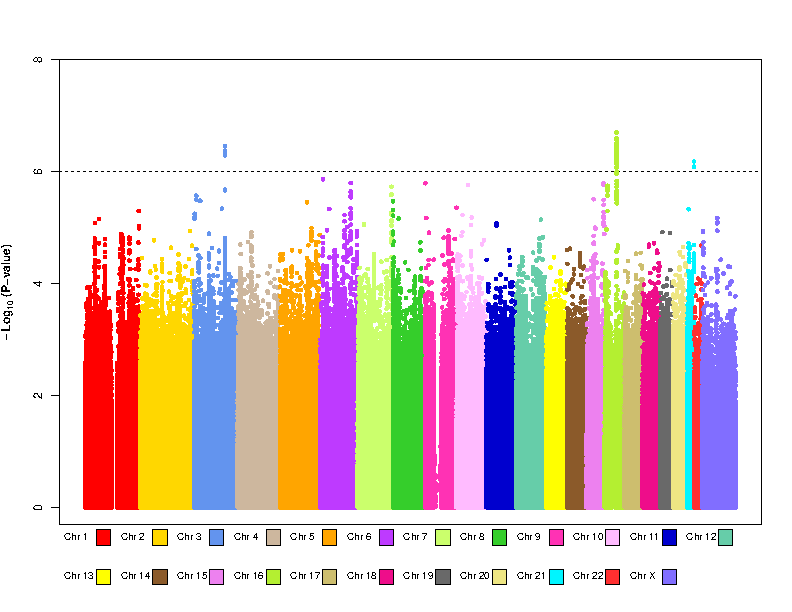

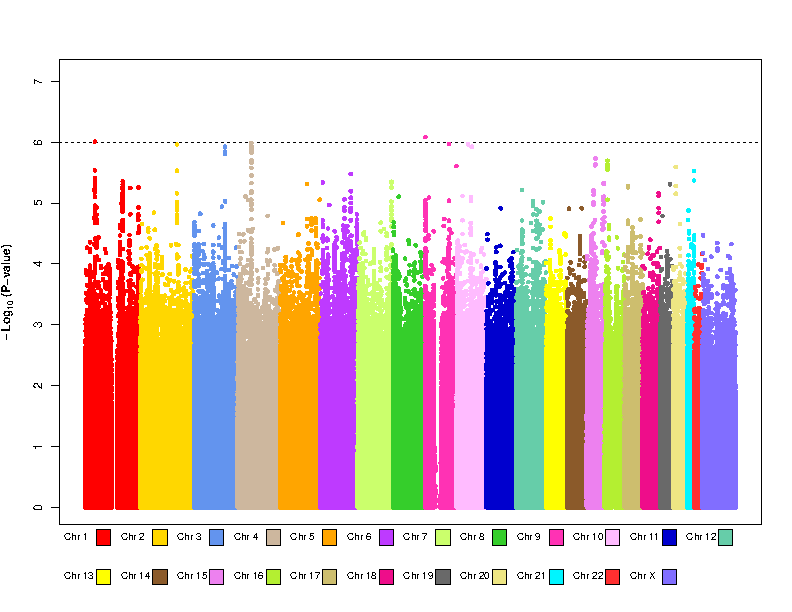

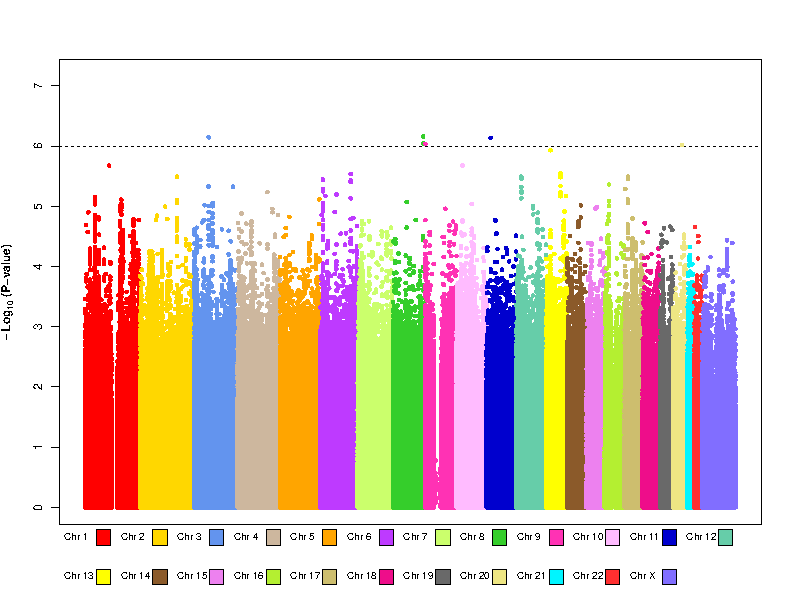

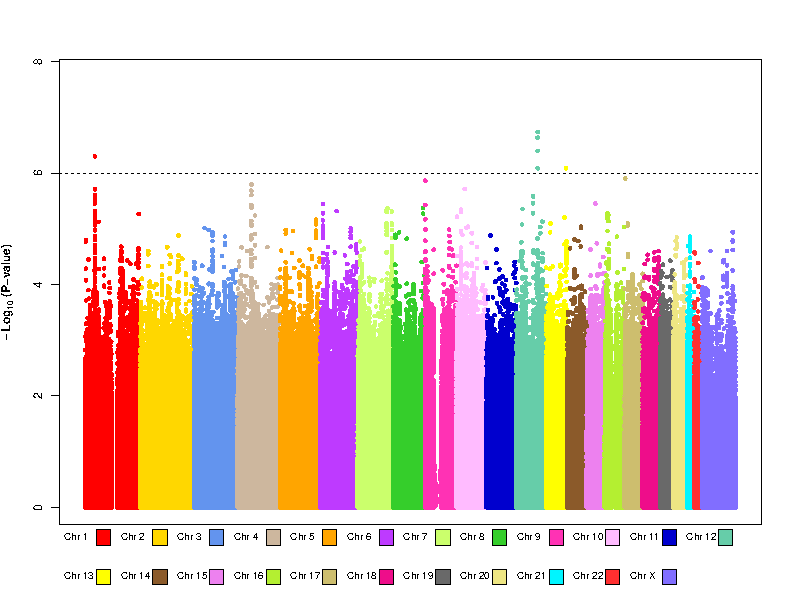

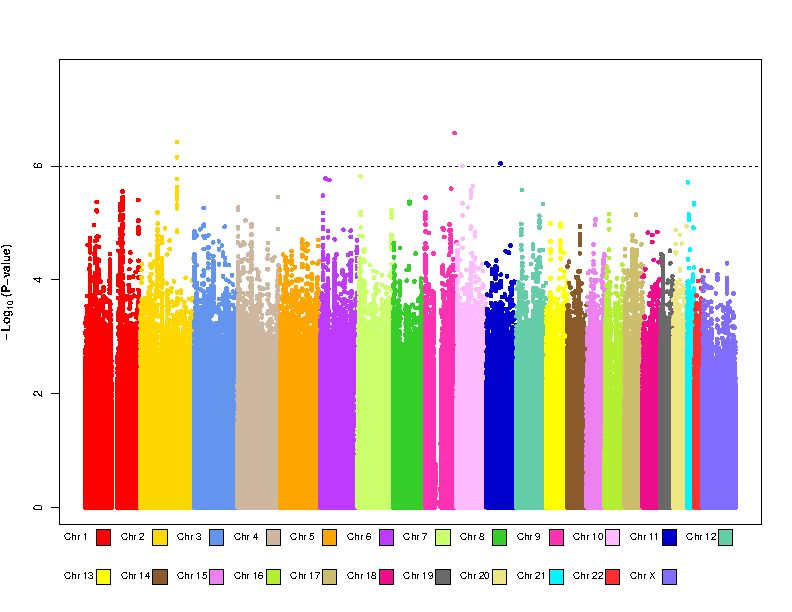


**c**

**
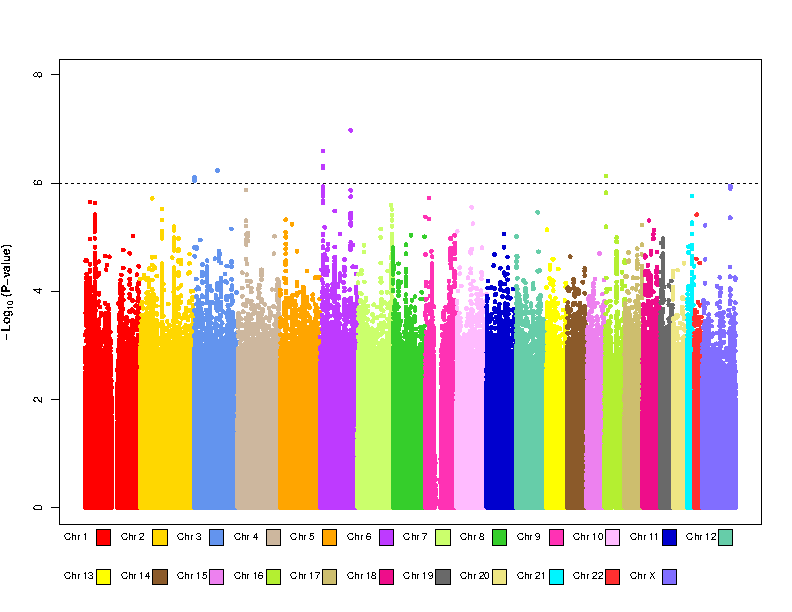

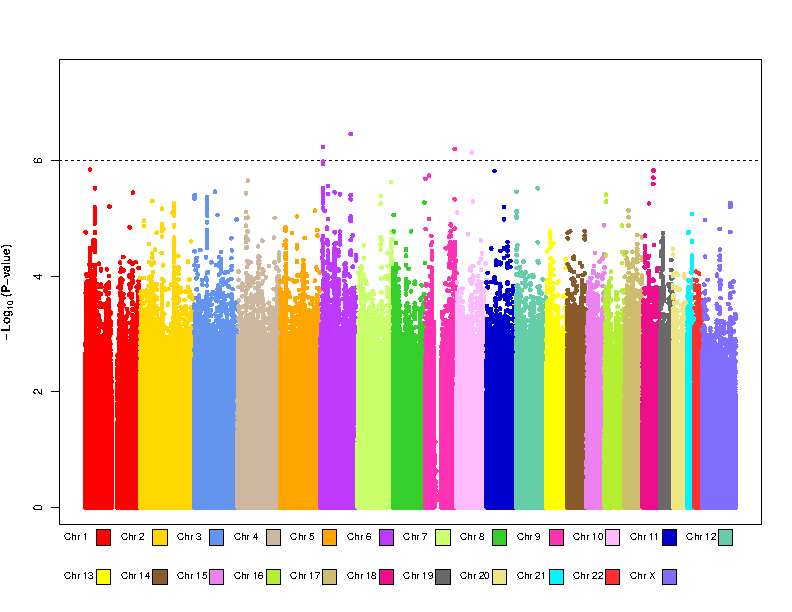

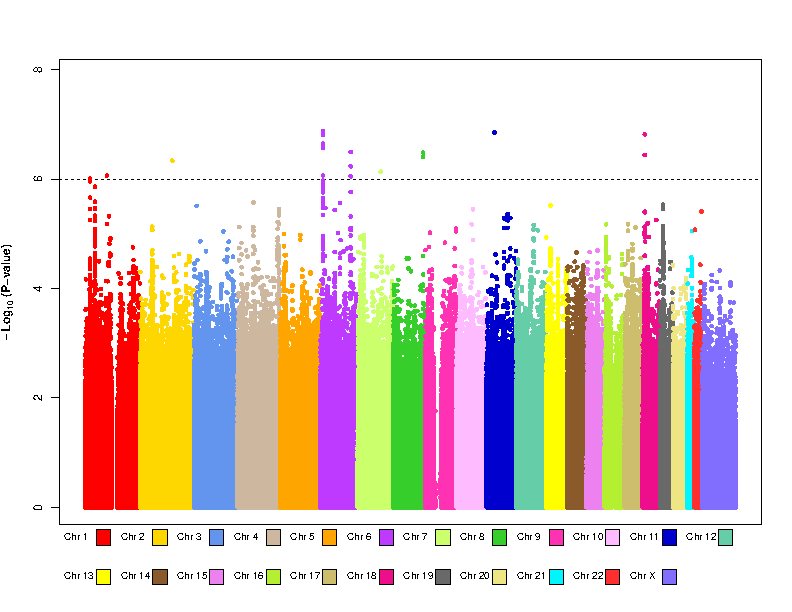

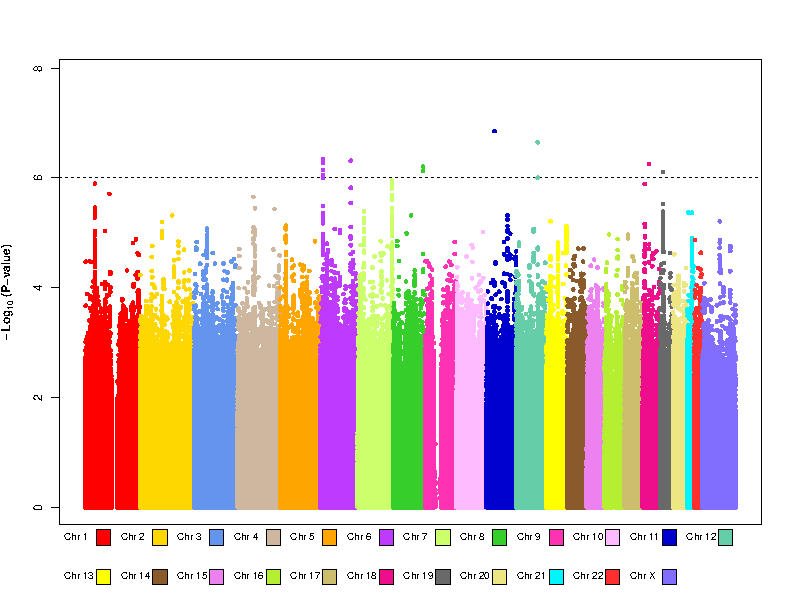

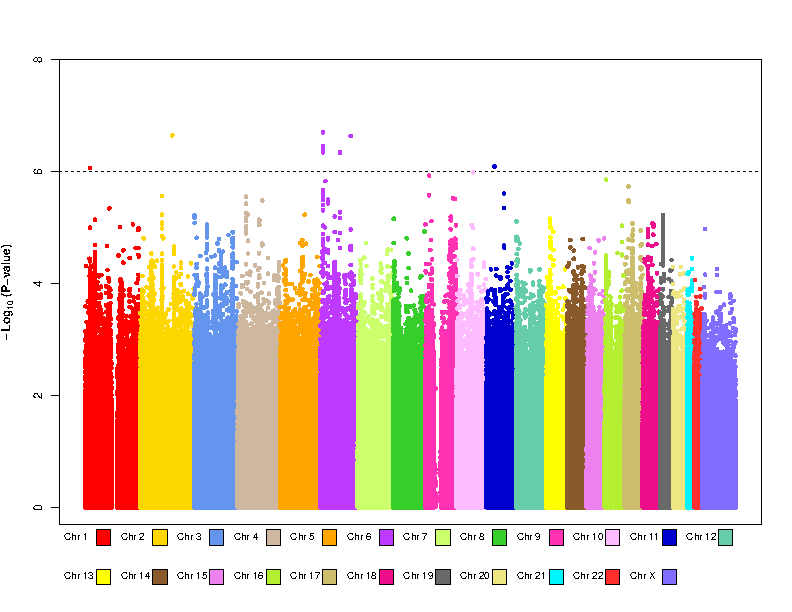

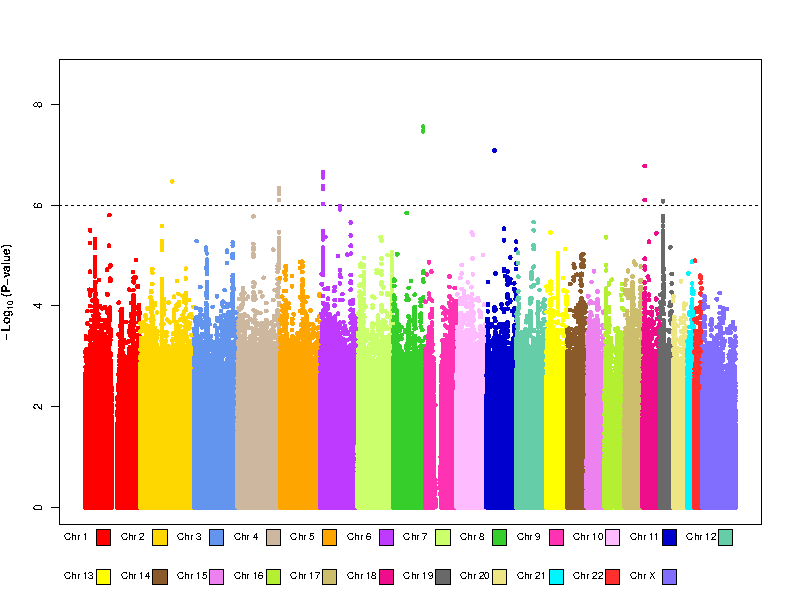
**

**d**

**
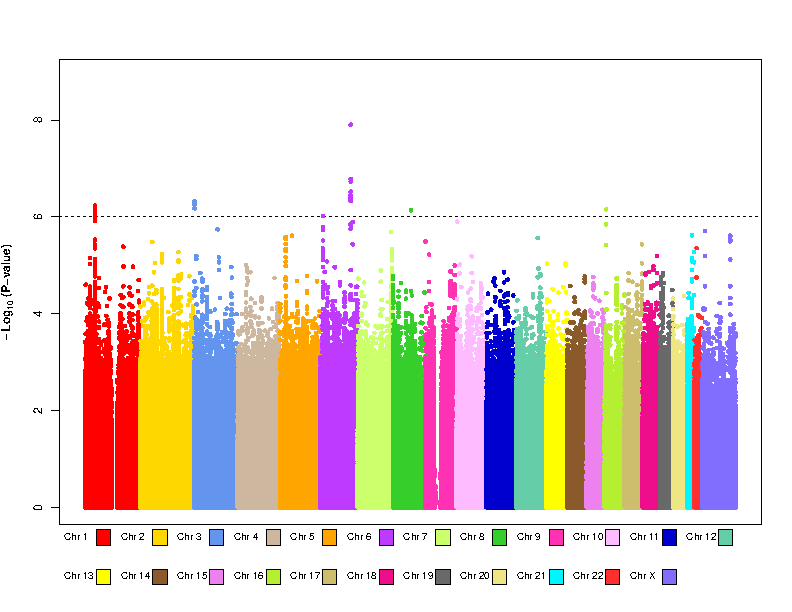

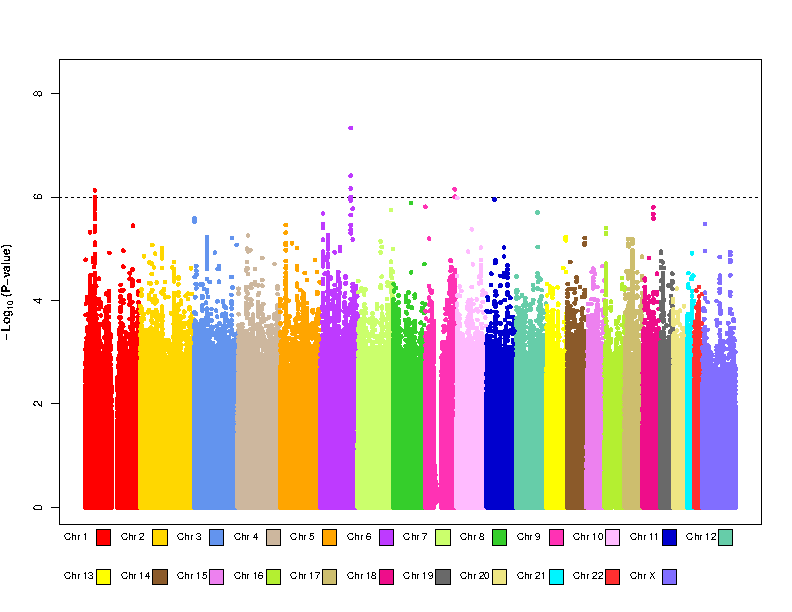

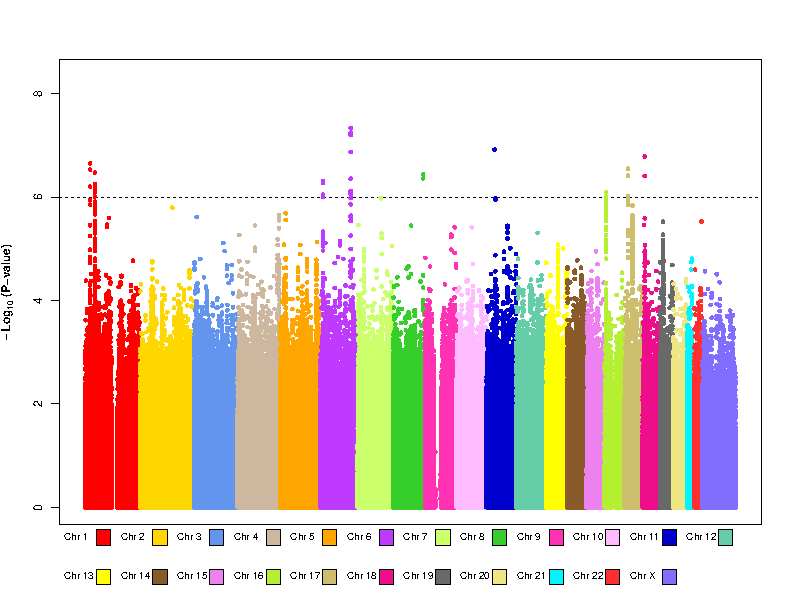

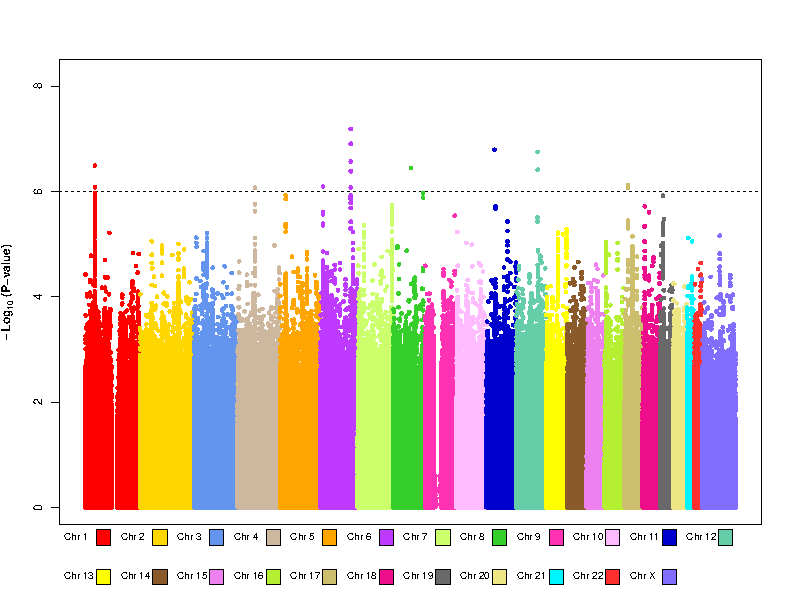

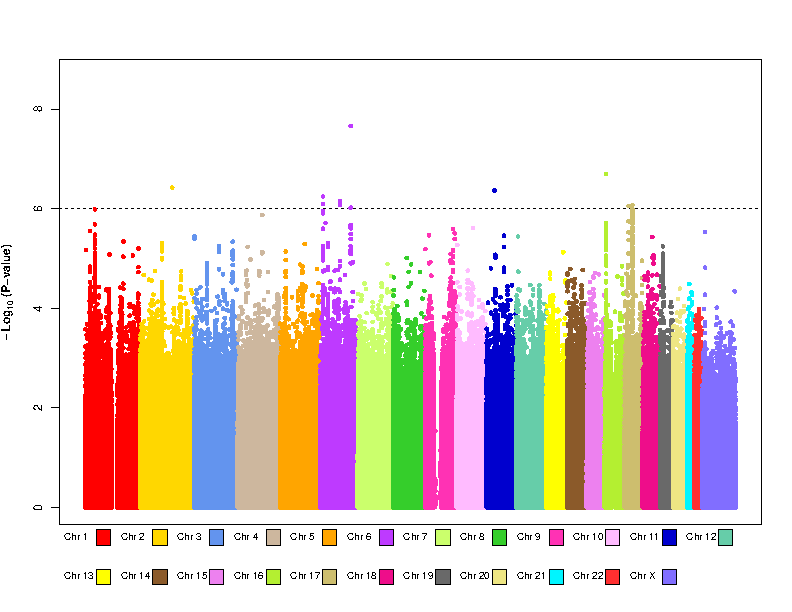

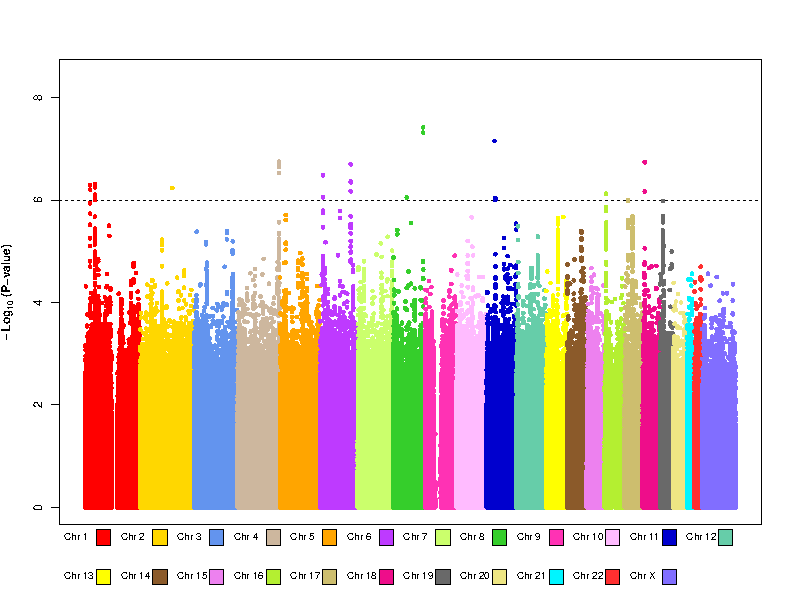
**

**e**


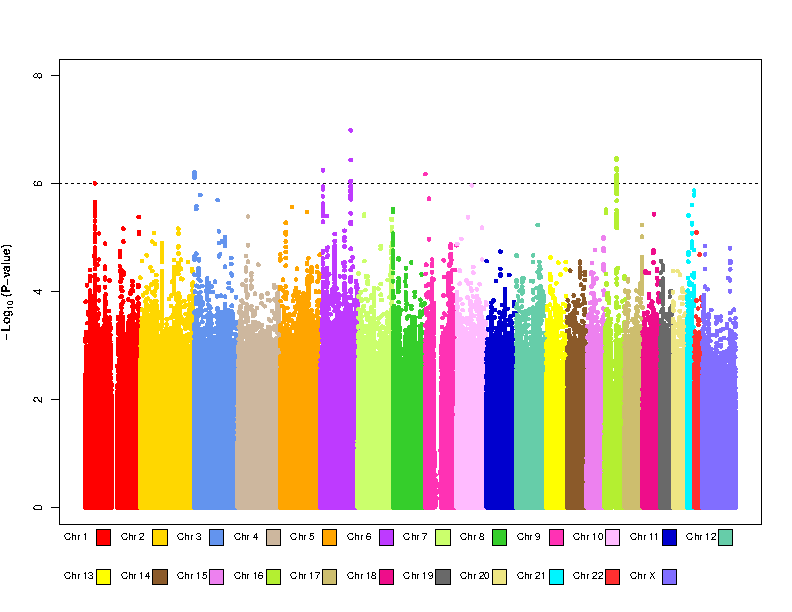

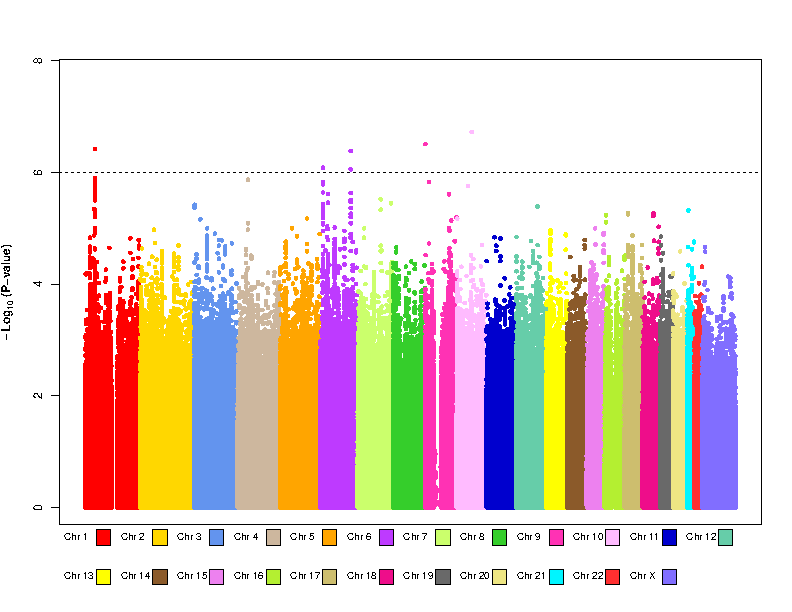

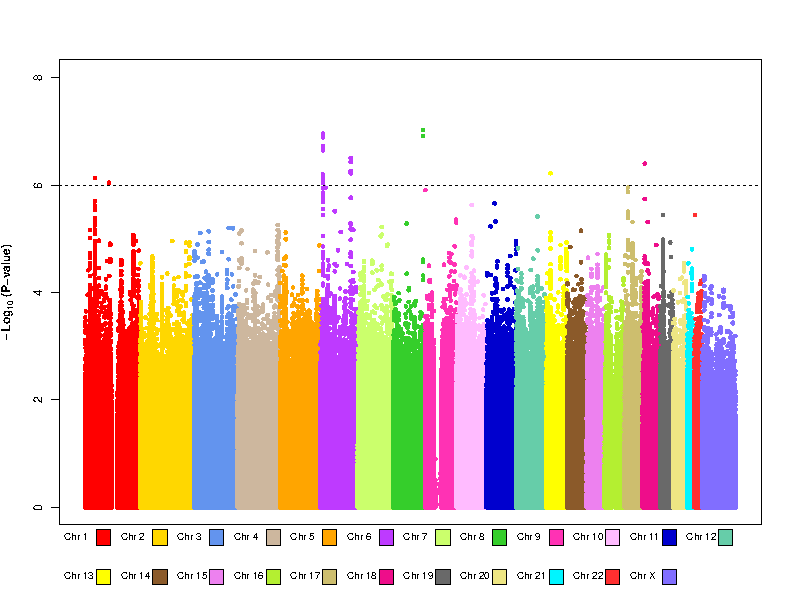

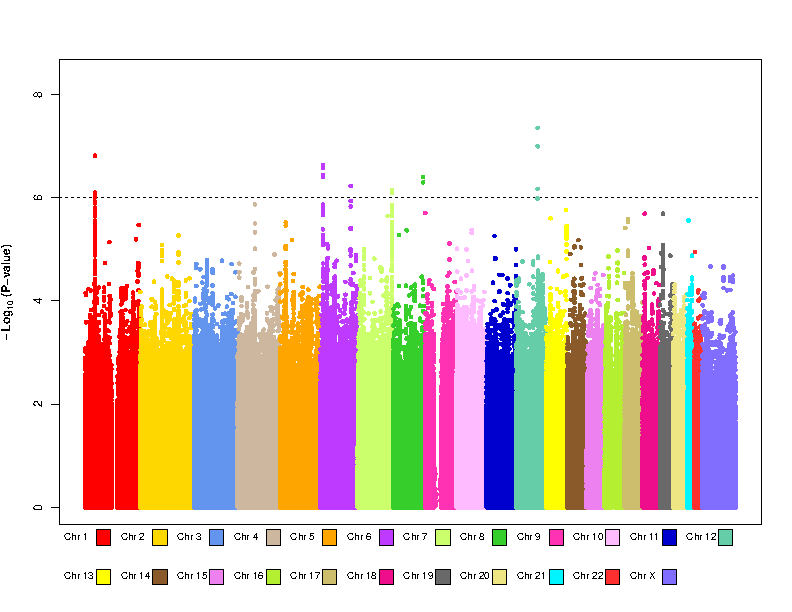

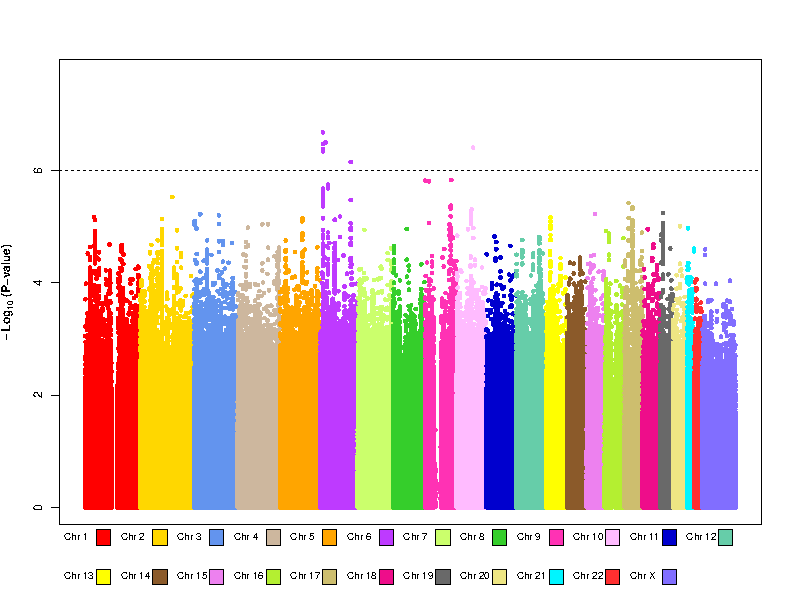

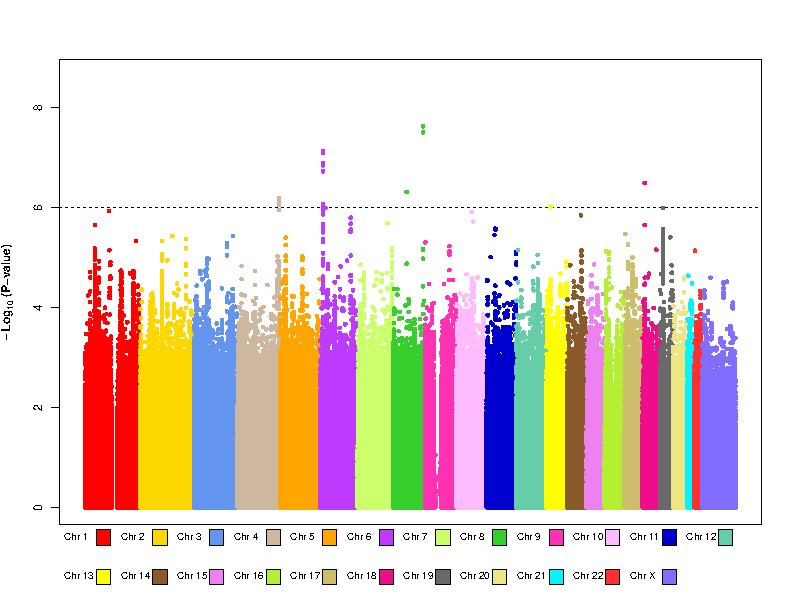


Manhattan plots of the GWAS meta-analysis results. **a** = V65L; **b** = V75L; **c** = V65H; **d** = V75H; **e** = VSUM. On each panel, the plots corresponding to Model 0 to Model 5 from left to right.

**Figure S4.** Quantile-quantile plots of the *P* value of heterogeneity

**a**


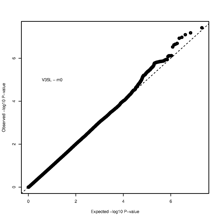

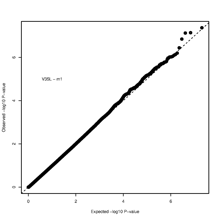

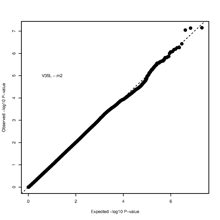

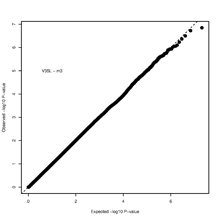

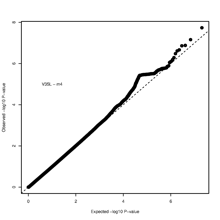

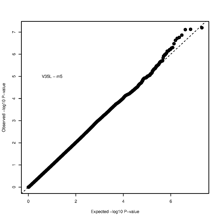


**b**

**
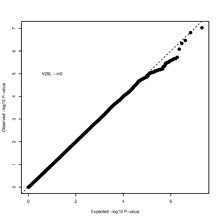

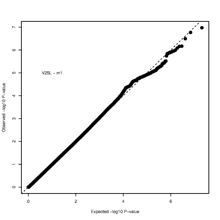

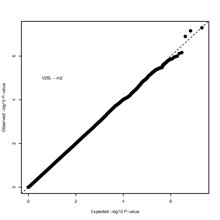

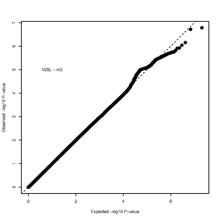

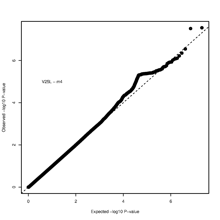

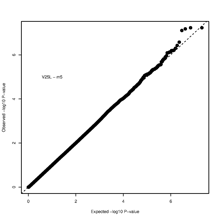
**

**c**

**
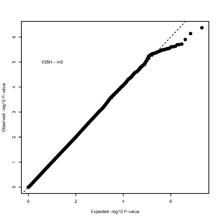

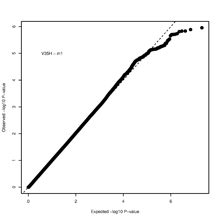

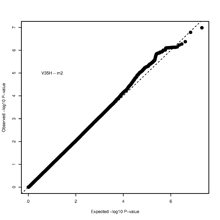

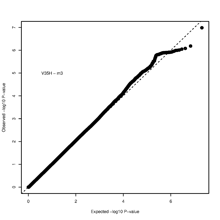

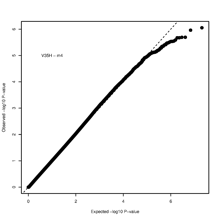

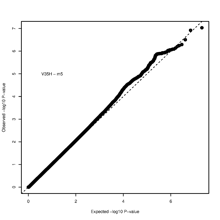
**

**d**

**
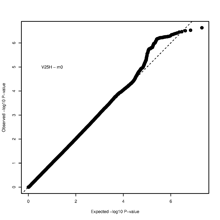

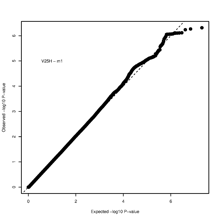

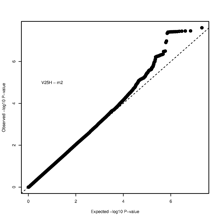

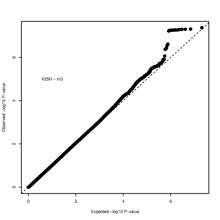

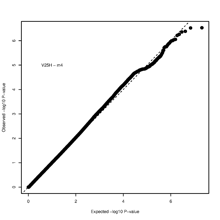

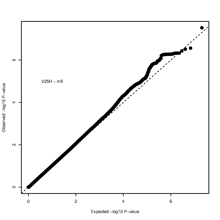
**

Quantile-quantile plots of the *P* value of heterogeneity. **a** = V65L; **b** = V75L; **c** = V65H; **d** = V75H. On each panel, the plots corresponding to Model 0 to Model 5 from left to right.

**Table S2.** Sources of summary statistics of breast cancer risk and breast cancer risk factors for calculating genetic correlation

| **Trait** | **Source** | **URL** |
| --- | --- | --- |
| Dense area | Chen et al. [1] | <https://bcac.ccge.medschl.cam.ac.uk/bcacdata/oncoarray/oncoarray-and-combined-summary-result/gwas-summary-results-mammographic-density-2021/> |
| Nondense area | Chen et al. [1] | <https://bcac.ccge.medschl.cam.ac.uk/bcacdata/oncoarray/oncoarray-and-combined-summary-result/gwas-summary-results-mammographic-density-2021/> |
| Percent density | Chen et al. [1] | <https://bcac.ccge.medschl.cam.ac.uk/bcacdata/oncoarray/oncoarray-and-combined-summary-result/gwas-summary-results-mammographic-density-2021/> |
| Overall breast cancer | Michailidou et al. [2] | <http://bcac.ccge.medschl.cam.ac.uk/bcacdata/oncoarray/oncoarray-and-combined-summary-result/gwas-summary-results-breast-cancer-risk-2017/> |
| ER+ breast cancer | Michailidou et al. [2] | <http://bcac.ccge.medschl.cam.ac.uk/bcacdata/oncoarray/oncoarray-and-combined-summary-result/gwas-summary-results-breast-cancer-risk-2017/> |
| ER− breast cancer | Michailidou et al. [2] | <http://bcac.ccge.medschl.cam.ac.uk/bcacdata/oncoarray/oncoarray-and-combined-summary-result/gwas-summary-results-breast-cancer-risk-2017/> |
| Adult body mass index | Pulit et al. [3] | <https://zenodo.org/record/1251813#.YPCd1xNKiSg> |
| Childhood body fatness | Warner et al. [4] | Not publicly available |
| Age at menarche | Day et al. [5] | <https://www.reprogen.org/data_download.html> |
| Age at natural menopause | Day et al. [6] | <https://www.reprogen.org/data_download.html> |

**Table S3.** Sources of summary statistics of breast cancer risk and breast cancer risk factors for the SNP-set test

| **Trait** | **Source** | **Number of SNPs included** |
| --- | --- | --- |
| Dense area | Sieh et al. [7] and Chen et al. [1] | 31 |
| Nondense area | Sieh et al. [7] and Chen et al. [1] | 16 |
| Percent density | Sieh et al. [7] and Chen et al. [1] | 23 |
| Overall breast cancer | Michailidou et al. [2] and Zhang et al. [8] | 178 |
| ER+ breast cancer | Michailidou et al. [2] and Zhang et al. [8] | 101 |
| ER− breast cancer | Michailidou et al. [2] and Zhang et al. [8] | 27 |
| Adult body mass index | Pulit et al. [3] | 279 |
| Childhood body fatness | Warner et al. [4] | 18 |
| Age at menarche | Day et al. [5] | 377 |
| Age at natural menopause | Day et al. [6] | 54 |

**References**

1. Chen H, Fan S, Stone J, Thompson DJ, Douglas J, Li S, Scott C, Bolla MK, Wang Q, Dennis J *et al*: **Genome-wide and transcriptome-wide association studies of mammographic density phenotypes reveal novel loci**. *Breast Cancer Res* 2022, **24**(1):27.

2. Michailidou K, Lindstrom S, Dennis J, Beesley J, Hui S, Kar S, Lemacon A, Soucy P, Glubb D, Rostamianfar A *et al*: **Association analysis identifies 65 new breast cancer risk loci**. *Nature* 2017, **551**(7678):92-94.

3. Pulit SL, Stoneman C, Morris AP, Wood AR, Glastonbury CA, Tyrrell J, Yengo L, Ferreira T, Marouli E, Ji Y *et al*: **Meta-analysis of genome-wide association studies for body fat distribution in 694 649 individuals of European ancestry**. *Hum Mol Genet* 2019, **28**(1):166-174.

4. Warner ET, Rice MS, Zeleznik OA, Fowler EE, Murthy D, Vachon CM, Bertrand KA, Rosner BA, Heine J, Tamimi RM: **Automated percent mammographic density, mammographic texture variation, and risk of breast cancer: a nested case-control study**. *NPJ Breast Cancer* 2021, **7**(1):68.

5. Day FR, Thompson DJ, Helgason H, Chasman DI, Finucane H, Sulem P, Ruth KS, Whalen S, Sarkar AK, Albrecht E *et al*: **Genomic analyses identify hundreds of variants associated with age at menarche and support a role for puberty timing in cancer risk**. *Nat Genet* 2017, **49**(6):834-841.

6. Day FR, Ruth KS, Thompson DJ, Lunetta KL, Pervjakova N, Chasman DI, Stolk L, Finucane HK, Sulem P, Bulik-Sullivan B *et al*: **Large-scale genomic analyses link reproductive aging to hypothalamic signaling, breast cancer susceptibility and BRCA1-mediated DNA repair**. *Nat Genet* 2015, **47**(11):1294-1303.

7. Sieh W, Rothstein JH, Klein RJ, Alexeeff SE, Sakoda LC, Jorgenson E, McBride RB, Graff RE, McGuire V, Achacoso N *et al*: **Identification of 31 loci for mammographic density phenotypes and their associations with breast cancer risk**. *Nat Commun* 2020, **11**(1):5116.

8. Zhang H, Ahearn TU, Lecarpentier J, Barnes D, Beesley J, Qi G, Jiang X, O'Mara TA, Zhao N, Bolla MK *et al*: **Genome-wide association study identifies 32 novel breast cancer susceptibility loci from overall and subtype-specific analyses**. *Nat Genet* 2020, **52**(6):572-581.
